# The Effect of Lutein Supplementation on Blood Plasma Levels of Complement Factor D, C5a and C3d

**DOI:** 10.1371/journal.pone.0073387

**Published:** 2013-08-29

**Authors:** Yuan Tian, Aize Kijlstra, Rob L. P. van der Veen, Maria Makridaki, Ian J. Murray, Tos T. J. M. Berendschot

**Affiliations:** 1 University Eye Clinic, Maastricht, Maastricht, The Netherlands; 2 Faculty of Life Sciences, University of Manchester, Manchester, United Kingdom; Centre for Eye Research Australia, Australia

## Abstract

Lutein is selectively taken up by the primate retina and plays an important role as a filter for harmful blue light and as an antioxidant. Recent studies have shown that lutein has systemic anti-inflammatory properties. Dietary lutein has been associated with reduced circulating levels of inflammatory biomarkers such as CRP and sICAM. Whether lutein also affects activation of the complement system has not yet been addressed and was the purpose of the study described here. Seventy-two subjects with signs of early macular degeneration were randomly assigned to receive either a 10 mg lutein supplement or a placebo during one year. EDTA blood samples were collected at 0, 4, 8 and 12 months. Complement factor D (CFD), a rate limiting component of the alternative pathway of complement activation and the complement activation products C5a and C3d were determined in the plasma samples by ELISA. A significant 0.11 µg/ml monthly decrease in plasma CFD concentration was observed in the lutein group (p<0.001), resulting in a 51% decrease from 2.3 µg/ml at baseline to 1.0 µg/ml at 12 months. The C5a concentration showed a significant 0.063ng/ml monthly decrease in the lutein group (p<0.001) resulting in a 36% decrease from 2.2ng/ml at baseline to 1.6ng/ml at 12 months. The C3d concentration showed a significant 0.19µg/ml monthly decrease in the lutein group (p=0.004) that gave rise to a 9% decrease from 15.4µg/ml at baseline to 14.4µg/ml at 12 months. In the placebo group we found a significant 0.04 µg/ml monthly decrease in plasma CFD concentration, whereas no changes were observed for C5a and C3d. Lutein supplementation markedly decreases circulating levels of the complement factors CFD, C5a and C3d levels, which might allow a simple method to control this inflammatory pathway of the innate immune system.

## Introduction

Lutein selectively accumulates in the primate retina and as a major component of the macular pigment it plays an important role as a filter to prevent harmful effects of blue light [1]. It also protects the retina from oxidative damage by quenching free oxygen radicals [2]. Various studies have recently shown that lutein also has anti-inflammatory properties (for review see Kijlstra et al. [3]). Lutein administration has led to beneficial effects in various models of experimental inflammation such as endotoxin induced uveitis [4], experimental age related macular degeneration [5,6], retinal ischemia [7,8] and diabetic retinopathy [9–11]. An inverse relationship has been shown between serum lutein concentration and markers of inflammation such as CRP and soluble ICAM-1 [12,13]. Whether lutein can also affect the complement pathway is not yet known and was the purpose of our study. The complement system plays an important role in the defense against microbial pathogens, clearance of apoptotic cells and chemotaxis of inflammatory cells [14]. A great deal of interest has recently been directed towards the role of the complement system in various eye diseases including age-related macular degeneration (AMD). This is based on the fact that drusen in the retina contain complement products [15,16] and because one of the strongest genetic associations with AMD has been found in genes encoding for a protein of this system (CFH gene) [17]. Furthermore, recent findings showed an increase in the level of various complement components in the circulation of AMD patients providing evidence for a systemic inflammatory component to the disease pathogenesis [18–22]. In view of these latter findings we chose individuals with early signs of AMD for our lutein intervention study.

## Methods

### Study design

The current study was a research project carried out with stored blood samples that had been collected during a randomized, double-blind, placebo-controlled two-centre intervention study (RCT) that was carried out in Manchester, UK and Maastricht, The Netherlands. This RCT was registered as a clinical trial NCT01042860 at clinicaltrials.gov and primary outcomes and results were recently published elsewhere [23]. Subjects were randomly assigned to receive either a 10 mg lutein supplement or a placebo based on soy bean oil. The UK arm of the study was approved by the South Manchester Regional Ethical Committee and The Netherlands arm of the study was approved by the Medical Ethical Committee at the University Hospital of Maastricht. The study was undertaken in accordance with the Declaration of Helsinki and all patients provided written informed consent. Patients were aware of the fact that blood samples were stored for future anonymous research questions. Uniform procedures and management strategies were used in the two centers. An advertising campaign was conducted within the universities and in local papers in both centers. Responding potential participants were contacted by letter and telephone.

### Subjects

Men or women aged between 50 to 80 years who met the following inclusion criteria were eligible to participate in the study: AMD grade 0 to 4 in one eye, best corrected visual acuity (BCVA) of LogMAR of 0.5 or better and minimal cataract. Subjects were excluded if they had any ophthalmic disorder other than early signs of AMD. In particular diabetic retinopathy, optic atrophy, pigmentary abnormalities considered by a supervising ophthalmologist to be less typical of AMD and history of glaucoma. Subjects with a BMI over 30 or who were diabetic were excluded. Finally, any potential participant consuming supplements containing lutein, zeaxanthin or meso-zeaxanthin or who had taken any of these at least 12 months before the study was not enrolled.

### Randomization and masking

The supplement was manufactured for the study by Cognis GmbH (now BASF SE; Rheinpromenade 1, 40789 Monheim, Germany) in accordance with Good Manufacturing Practice. A randomization code was generated by the sample manufacturer and capsules were distributed in accordance with this list. Each patient was assigned a specific treatment number. Treatment numbers were allocated in ascending order using the next available consecutive number. If a discontinued patient was replaced, the next available treatment number was used. The code linking treatment number with capsule allocation remained with the manufacturer until the end of the intervention trial. The study personnel were unaware of which patients were assigned to which groups. The study products were packaged and delivered under the responsibility of Cognis. Each participant was allocated a box (a treatment unit) in which there were 14 white plastic cartons. Each of these held 36 capsules, sufficient for 1 month (31 days plus 5 extra). The placebo and lutein containers were indistinguishable. Each treatment unit represented the complete study product for one participant. It contained a total of 504 capsules, enough capsules for 12 months of treatment (365 days plus 139 more). The supplementation products were stored safely according to manufacturer’s guidelines at temperatures below 25 °C and not exposed to light. Four 3 monthly visits were scheduled at which four cartons (144 capsules) were given to each participant. The safety of the lutein supplement has been addressed elsewhere [24,25].

### Blood sampling and complement factor analysis

Blood samples were taken using EDTA containing tubes at the time of the scheduled visits. They were centrifuged within one hour and stored in aliquots at -80 degrees Celsius. CFD was measured at a 1/4000 dilution using a commercially available development kit (DuoSet) for human complement factor D (R&D Systems, Minneapolis, USA) according to the manufacturer’s instructions. C5a was measured at a 1/10 dilution using a commercially available development kit (DuoSet) for human complement component C5a (R&D Systems, Minneapolis, USA). C3d was measured at a 1/10 dilution using a development kit for human complement fragment C3d (MyBioSource，San Diego, USA).

### Statistical analysis

Statistical analysis was performed using SPSS 20.0.0. Differences in gender distribution over the experimental groups were tested using the Pearson Chi-square test, while differences in age and plasma concentrations between groups was evaluated using ANOVA. To quantify the course in time, a Linear Mixed Models analysis (LMM) was performed with subject ID as grouping factor, and gender, supplementation time, and the interaction term of the latter two as covariates. All statistical tests were done at a two-sided significance level of 0.05.

Data of the individual complement levels are available on request from the corresponding author.

## Results

Seventy-two subjects were randomly assigned to the two intervention groups. An extensive flow diagram of the included volunteers is shown in the paper published of the original RCT [23]. Table 1 shows the baseline characteristics of the 72 subjects. Age en gender as well as baseline plasma concentrations of CFD, C5a, and C3d were not statistically different among the two groups.

**Table 1:** Baseline characteristics for the lutein and placebo group (mean ± st dev).

	lutein	placebo	p-value
Gender (m/f)	15/20	14/23	0.81
Age (years)	72 ± 9	69 ± 9	0.14
CFD* (μg/ml)	2.3 ± 1.5	1.9 ± 1.3	0.27
C5a (ng/ml)	2.2 ± 0.7	2.21 ± 0.6	0.95
C3d (μg/ml)	15.4 ± 8.6	15.5 ± 7.2	0.99

* CFD: Complement Factor D

**Figure 1:**
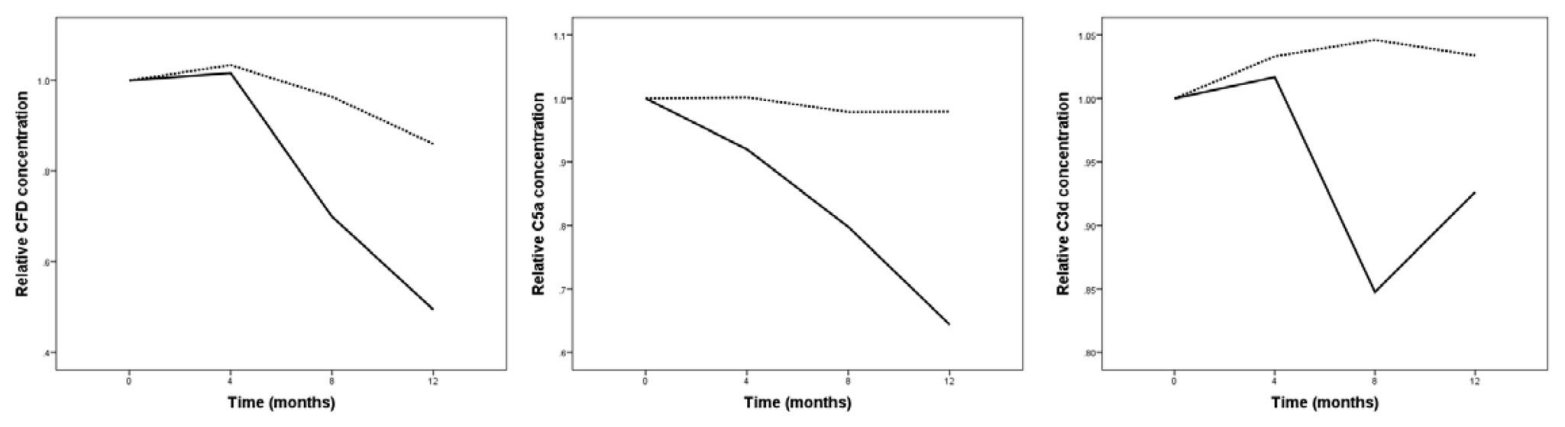
Mean relative plasma concentration of complement factor D (CFD) (left), C5a concentration (middle) and C3d (right) in time for the lutein (solid line) and placebo group (dotted line). Values at baseline were taken as the reference, i.e. 1.0.

Following supplementation with a daily dose of 10 mg lutein, all measured complement factors (CFD, C5a and C3d) decreased over time in the lutein group (Table 2). In the placebo group, C3d and C5a levels remained constant over time, whereas the CFD levels showed a decrease in time. Separate analysis of the values from each individual relative to the baseline concentration showed a decrease of 51%, 36% and 8% for CFD, C5a and C3d respectively in the lutein group. In the placebo group, CFD showed a 14% decrease, whereas no significant changes were observed for C5a and C3d (see Figure 1).

**Table 2:** Plasma Complement levelsover time.

		baseline			4 months			8 months			12 months		
CFD^*^ (μg/ml)	Lutein	2.3	±	1.5	1.8	±	1.0	1.2	±	0.7	1.0	±	0.5
	Placebo	1.9	±	1.3	1.6	±	1.4	1.4	±	1.3	1.4	±	1.0
C5a (ng/ml)	Lutein	2.2	±	0.7	2.1	±	0.7	1.8	±	0.8	1.6	±	0.8
	Placebo	2.2	±	0.6	2.1	±	0.8	2.1	±	0.6	2.2	±	0.7
C3d (μg/ml)	Lutein	15.4	±	8.6	15.8	±	10.1	14.3	±	9.4	14.4	±	10.6
	Placebo	15.5	±	7.2	15.0	±	6.8	14.4	±	6.8	16.0	±	7.9

* CFD: Complement Factor D

Linear Mixed Model (LMM) analysis revealed a significant effect for time (p<0.001), as well as the interaction between supplementation and time (p=0.006) on CFD plasma levels. Levels of significance for the time effect for plasma C5a and C3d were p<0.001 and p=0.057. For the interaction between supplementation and time these values were p<0.001 and p=0.010 for C5a and C3d, respectively.

Stratifying for supplementation we found a significant 0.11 µg/ml monthly decrease in plasma CFD concentration in the lutein group (p<0.001), resulting in a decrease from 2.3 µg/ml at baseline to 1.0 µg/ml at 12 months. The C5a concentration showed a significant 0.063ng/ml monthly decrease in the lutein group (p<0.001) resulting in a decrease from 2.2ng/ml at baseline to 1.6ng/ml at 12 months. The C3d concentration showed a significant 0.19µg/ml monthly decrease in the lutein group (p=0.004) that gave rise to a decrease from 15.4µg/ml at baseline to 14.4µg/ml at 12 months. In the placebo group we found a significant 0.04 µg/ml monthly decrease in plasma CFD concentration. No significant changes were observed for C5a and C3b. We incorporated gender as covariate into the LMM analysis, but did not find a significant effect of gender on the effect of supplementation on plasma concentrations of the complement factors. We also found no difference between the two participating centers.

**Figure 2:**
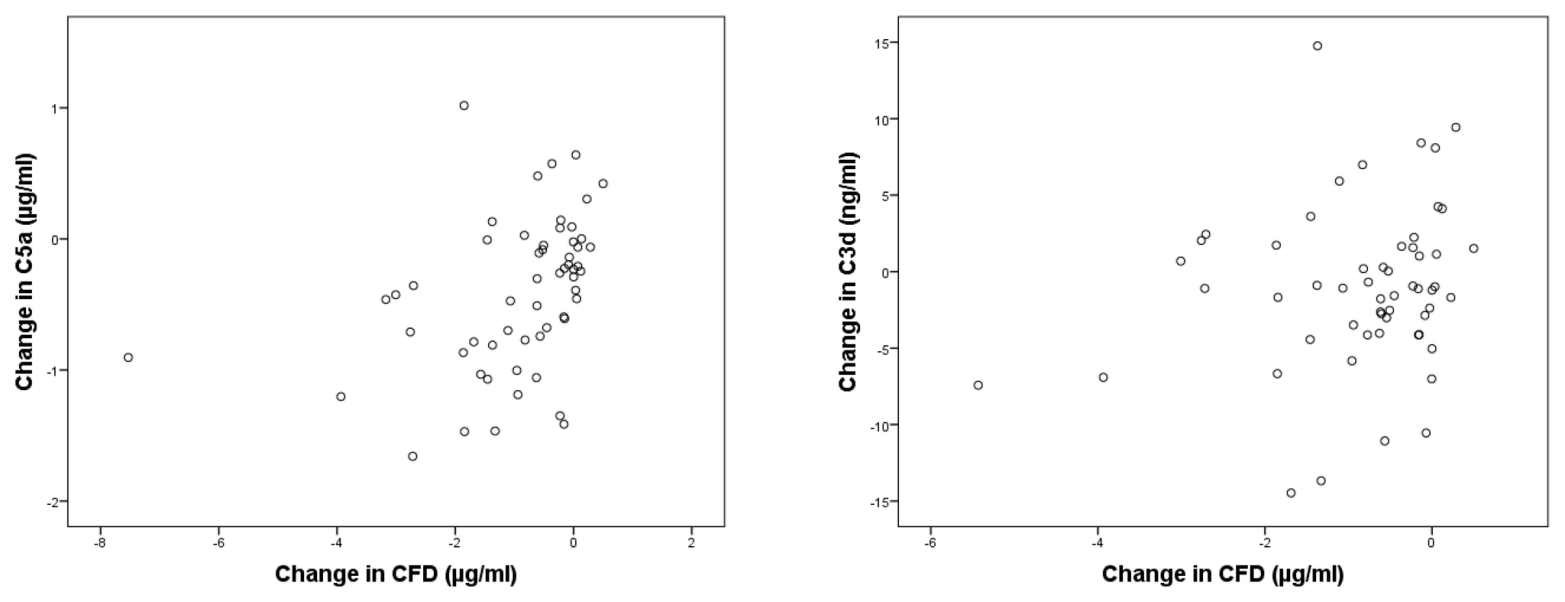
Change in serum concentration from baseline to 12 months for C5a (left) and C3d (right) as a function of the change in CFD concentration from baseline to 12 months (r=0.37, p=0.004 and r=0.18, p=0.16 respectively).

Figure 2 shows the change in serum C5a and C3d concentration from baseline to 12 months as a function of the change in CFD concentration from baseline to 12 months (r=0.37, p = 0.004and r=0.19, p=0.16 respectively). A LLM analysis whereby either C5a or C3d plasma levels were taken as dependent, subject ID as grouping factor and CFD plasma levels as covariates yielded a significant association between plasma C5a and CFD concentration (β=0.090, p=0.006). For C3d no significant association was observed.

## Discussion

This study shows for the first time that daily supplementation with lutein can decrease the circulating levels of the complement factors CFD, C3d and C5a in an elderly population.

At baseline we found a mean CFD plasma concentration of 2.09± 1.43 µg/ml. This is in between the median values of 2.31µg/ml for AMD subjects and 2.08 µg/ml in controls as recently reported by others using the same ELISA kit [21]. Using a different kit to determine CFD, two other studies found somewhat lower values: 1.26 µg/ml for AMD subjects and 0.95 µg/ml for controls [18] and 1.50 µg/ml for AMD subjects and 1.16 µg/ml for controls [22]. The mean C5a concentration at baseline was 2.21ng/ml in this study. This is in between the median values of 1.85ng/ml and 4.28 ng/m in AMD subjects and 1.67 ng/ml and 4.06 ng/ml in controls as recently reported by others using a different kit [18,22]. The mean C3d concentration at baseline was 15.4µg/ml in our study. This is lower than median values of 55.2 µg/ml and 40.7 µg/ml in AMD subjects and 46.9 ng/ml and 35.8 µg/ml in controls as recently reported by others using a different kit [18,22]. Note that these studies all showed raised levels of the complement factors in AMD subjects as compared to controls pointing to a role in AMD pathogenesis.

A deregulated control of the alternative pathway of the complement system has been shown to play an important role in the pathogenesis of AMD [17]. CFD is a key enzyme in this pathway, resulting in the assembly of the alternative pathway C3 convertase and controlling the subsequent steps leading to important inflammatory activation products such as C3a and C5a [26,27]. CFD is the rate limiting enzyme in the activation sequence of the alternative pathway and its level in blood are quite low. The limitation is overcome at 9-10 times the normal physiological level [28]. Small alterations of the CFD level will thus have profound effects on the activation of the alternative pathway of complement. In this study we found a 51% decrease in CFD and an accompanying 36% decrease in C5a, in accordance with this concept. It was further confirmed by the fact that we found a positive correlation (r=0.37, p=0.004) between the change in CFD and the change in C5a (see Figure 2) and the fact that a LMM analysis showed a significant association between plasma CFD and C5a concentration (β=0.090, p=0.006). C3d only showed a small but significant 9% decrease, and the association with CFD changes was less clear. This may be due to the fact that C3d,which is formed following decay of C3b, is mainly present on the activating surface and only small amounts are generated in the fluid phase.

We thus hypothesize that lutein supplementation decreases CFD levels, which as a result will lower complement activation products downstream in the complement cascade. CFD, also known as adipsin, is mainly produced by adipocytes [28,29]. Its low blood level is caused by an extremely high catabolic rate and loss by glomerular filtration. Whether lutein would affect production or catabolism and whether other carotenoids have the same effect remains an open question. Of interest is the fact that adipocytes are the main storage site for carotenoids including lutein [30]. A literature search failed to find studies linking lutein in adipocytes and whether it could affect the production of CFD.

A small but significant decrease in CFD levels was also found in the placebo group. Before entering the trial subjects were informed about the nature and background of the study. This may have led to a change in their dietary habits. It could also be due to a seasonal effect, but analysis of the dates that subjects entered the trial did not support this explanation. We also investigated CRP levels in plasma over time but did not find a significant change either in the treatment or placebo group (data not shown). A phase I/II clinical trial was recently started to lower CFD in the eye itself. In this trial the effect of an intravitreal injection of a monoclonal antibody against CFD (FCFD4514S) is being evaluated for the treatment of geographic atrophy (Genentech, San Francisco, USA, ClinicalTrials.gov NCT01229215). A much more appealing option would be to control CFD levels via the diet, as put forward by our study.

The role of nutrition in the prevention and progression of AMD has received a great deal of attention over the past decades. Studies on nutritional modulation of AMD were recently reviewed by Weikel and Taylor [31]. They suggested that 10 mg of lutein, the amount also provided in this study, confers the highest benefit to the health of the retina.

There are a number of limitations in our study. It had a relatively small sample size and we did not equalize for possible confounders such as dieter health of the participants. We did exclude individuals with diabetes or with a BMI above 30. Other possible confounders such as weight changes during the study, the presence of chronic (inflammatory) disease in the study group which has an age over 65, use of anti-inflammatory medication, total energy intake, physical activity or smoking were not recorded. In the trial we replaced discontinuing subjects with new ones. The reasons for discontinuation and those lost to follow up were described in detail in our earlier paper on this study [23]. It might have resulted in unequal distributions between treatment and control groups, but this could not be evaluated because data on diet, lifestyle or medication which might have an influence on blood complement levels were not collected and compared. Larger trials are needed in the future to confirm the present observations and to rule out the possible confounding factors listed above.

In conclusion, due to the key role of CFD in the activation pathway of the alternative pathway of the complement system and the potentially profound effect of lutein on circulating levels of CFD, this study suggests that lutein supplementation may be a powerful tool in the prevention and treatment of AMD, although further studies to replicate the significance of these findings are now clearly warranted.

## References

[1] Landrum JT, Bone RA, Kilburn MD (1997) The macular pigment: a possible role in protection from age-related macular degeneration. Adv Pharmacol 38: 537-556. PubMed: 8895823. 8895823 10.1016/s1054-3589(08)60998-9

[2] Khachik F, Bernstein PS, Garland DL (1997) Identification of lutein and zeaxanthin oxidation products in human and monkey retinas. Invest Ophthalmol Vis Sci 38: 1802-1811. PubMed: 9286269. 9286269

[3] Kijlstra A, Tian Y, Kelly ER, Berendschot TTJM (2012) Lutein: more than just a filter for blue light. Prog Retin Eye Res 31: 303-315. doi:10.1016/j.preteyeres.2012.03.002. PubMed: 22465791. 22465791 10.1016/j.preteyeres.2012.03.002

[4] Jin XH, Ohgami K, Shiratori K, Suzuki Y, Hirano T et al. (2006) Inhibitory effects of lutein on endotoxin-induced uveitis in Lewis rats. Invest Ophthalmol Vis Sci 47: 2562-2568. doi:10.1167/iovs.05-1429. PubMed: 16723471. 16723471 10.1167/iovs.05-1429

[5] Izumi-Nagai K, Nagai N, Ohgami K, Satofuka S, Ozawa Y et al. (2007) Macular Pigment Lutein Is Antiinflammatory in Preventing Choroidal Neovascularization. Arterioscler Thromb Vasc Biol 27: 2555-2562. doi:10.1161/ATVBAHA.107.151431. PubMed: 17932319. 17932319 10.1161/ATVBAHA.107.151431

[6] Kijlstra A, La Heij EC, Hendrikse F (2005) Immunological factors in the pathogenesis and treatment of age-related macular degeneration. Ocul Immunol Inflamm 13: 3-11. doi:10.1080/09273940590909185. PubMed: 15804763. 15804763 10.1080/09273940590909185

[7] Li SY, Fu ZJ, Ma H, Jang WC, So KF et al. (2009) Effect of lutein on retinal neurons and oxidative stress in a model of acute retinal ischemia/reperfusion. Invest Ophthalmol Vis Sci 50: 836-843. PubMed: 18936152. 18936152 10.1167/iovs.08-2310

[8] Li SY, Lo AC (2010) Lutein Protects RGC-5 Cells Against Hypoxia and Oxidative Stress. Int J Mol Sci 11: 2109-2117. doi:10.3390/ijms11052109. PubMed: 20559505. 20559505 10.3390/ijms11052109 PMC2885097

[9] Muriach M, Bosch-Morell F, Alexander G, Blomhoff R, Barcia J et al. (2006) Lutein effect on retina and hippocampus of diabetic mice. Free Radic Biol Med 41: 979-984. doi:10.1016/j.freeradbiomed.2006.06.023. PubMed: 16934681. 16934681 10.1016/j.freeradbiomed.2006.06.023

[10] Sasaki M, Ozawa Y, Kurihara T, Kubota S, Yuki K et al. (2010) Neurodegenerative influence of oxidative stress in the retina of a murine model of diabetes. Diabetologia 53: 971-979. doi:10.1007/s00125-009-1655-6. PubMed: 20162412. 20162412 10.1007/s00125-009-1655-6 PMC2850533

[11] Brazionis L, Rowley K, Itsiopoulos C, O’Dea K (2009) Plasma carotenoids and diabetic retinopathy. Br J Nutr 101: 270-277. doi:10.1017/S0007114508006545. PubMed: 18554424. 18554424 10.1017/S0007114508006545

[12] van Herpen-Broekmans WM, Klöpping-Ketelaars IA, Bots ML, Kluft C, Princen H et al. (2004) Serum carotenoids and vitamins in relation to markers of endothelial function and inflammation. Eur J Epidemiol 19: 915-921. doi:10.1007/s10654-004-5760-z. PubMed: 15575349. 15575349 10.1007/s10654-004-5760-z

[13] Seddon JM, Gensler G, Klein ML, Milton RC (2006) C-reactive protein and homocysteine are associated with dietary and behavioral risk factors for age-related macular degeneration. Nutrition 22: 441-443. doi:10.1016/j.nut.2005.12.004. PubMed: 16530626. 16530626 10.1016/j.nut.2005.12.004

[14] Sjöberg AP, Trouw LA, Blom AM (2009) Complement activation and inhibition: a delicate balance. Trends Immunol 30: 83-90. doi:10.1016/j.it.2008.11.003. PubMed: 19144569. 19144569 10.1016/j.it.2008.11.003

[15] van der Schaft TL, Mooy CM, de Bruijn WC, de Jong PTVM (1993) Early stages of age-related macular degeneration: an immunofluorescence and electron microscopy study. Br J Ophthalmol 77: 657-661. doi:10.1136/bjo.77.10.657. PubMed: 8218037. 8218037 10.1136/bjo.77.10.657 PMC504611

[16] Hageman GS, Luthert PJ, Chong NHV, Johnson LV, Anderson DH et al. (2001) An integrated hypothesis that considers drusen as biomarkers of immune-mediated processes at the RPE-Bruch’s membrane interface in aging and age-related macular degeneration. Prog Retin Eye Res 20: 705-732. doi:10.1016/S1350-9462(01)00010-6. PubMed: 11587915. 11587915 10.1016/s1350-9462(01)00010-6

[17] Anderson DH, Radeke MJ, Gallo NB, Chapin EA, Johnson PT et al. (2010) The pivotal role of the complement system in aging and age-related macular degeneration: hypothesis re-visited. Prog Retin Eye Res 29: 95-112. doi:10.1016/j.preteyeres.2009.11.003. PubMed: 19961953. 19961953 10.1016/j.preteyeres.2009.11.003 PMC3641842

[18] Scholl HP, Charbel Issa P, Walier M, Janzer S, Pollok-Kopp B et al. (2008) Systemic complement activation in age-related macular degeneration. PLOS ONE 3: e2593. doi:10.1371/journal.pone.0002593. PubMed: 18596911. 18596911 10.1371/journal.pone.0002593 PMC2440421

[19] Reynolds R, Hartnett ME, Atkinson JP, Giclas PC, Rosner B et al. (2009) Plasma Complement Components and Activation Fragments: Associations with Age-Related Macular Degeneration Genotypes and Phenotypes. Invest Ophthalmol Vis Sci 50: 5818-5827. doi:10.1167/iovs.09-3928. PubMed: 19661236. 19661236 10.1167/iovs.09-3928 PMC2826794

[20] Smailhodzic D, Klaver CC, Klevering BJ, Boon CJ, Groenewoud JM et al. (2012) Risk alleles in CFH and ARMS2 are independently associated with systemic complement activation in age-related macular degeneration. Ophthalmologe 119: 339-346. doi:10.1016/j.ophtha.2011.07.056. PubMed: 22133792. 10.1016/j.ophtha.2011.07.056 22133792

[21] Stanton CM, Yates JRW, den Hollander AI, Seddon JM, Swaroop A et al. (2011) Complement factor D in age-related macular degeneration. Invest Ophthalmol Vis Sci 52: 8828-8834. doi:10.1167/iovs.11-7933. PubMed: 22003108. 22003108 10.1167/iovs.11-7933 PMC3230905

[22] Hecker LA, Edwards AO, Ryu E, Tosakulwong N, Baratz KH et al. (2010) Genetic control of the alternative pathway of complement in humans and age-related macular degeneration. Hum Mol Genet 19: 209-215. doi:10.1093/hmg/ddp472. PubMed: 19825847. 19825847 10.1093/hmg/ddp472 PMC2792151

[23] Murray IJ, Makridaki M, van der Veen RLP, Carden D, Parry NR et al. (2013) Lutein Supplementation over a One-Year Period in Early AMD Might Have a Mild Beneficial Effect on Visual Acuity: The CLEAR Study. Invest Ophthalmol Vis Sci 54: 1781-1788. doi:10.1167/iovs.12-10715. PubMed: 23385792. 23385792 10.1167/iovs.12-10715

[24] Connolly EE, Beatty S, Loughman J, Howard AN, Louw MS et al. (2011) Supplementation with all three macular carotenoids: response, stability, and safety. Invest Ophthalmol Vis Sci 52: 9207-9217. doi:10.1167/iovs.11-8025. PubMed: 21979997. 21979997 10.1167/iovs.11-8025

[25] Ravikrishnan R, Rusia S, Ilamurugan G, Salunkhe U, Deshpande J et al. (2011) Safety assessment of lutein and zeaxanthin (Lutemax 2020): subchronic toxicity and mutagenicity studies. Food Chem Toxicol 49: 2841-2848. doi:10.1016/j.fct.2011.08.011. PubMed: 21872637. 21872637 10.1016/j.fct.2011.08.011

[26] Forneris F, Ricklin D, Wu J, Tzekou A, Wallace RS et al. (2010) Structures of C3b in complex with factors B and D give insight into complement convertase formation. Science 330: 1816-1820. doi:10.1126/science.1195821. PubMed: 21205667. 21205667 10.1126/science.1195821 PMC3087196

[27] Katschke KJ, Wu P, Ganesan R, Kelley RF, Mathieu MA et al. (2012) Inhibiting Alternative Pathway Complement Activation by Targeting the Factor D Exosite. J Biol Chem 287: 12886-12892. doi:10.1074/jbc.M112.345082. PubMed: 22362762. 22362762 10.1074/jbc.M112.345082 PMC3339934

[28] Volanakis JE, Narayana SVL (1996) Complement factor D, a novel serine protease. Protein Sci 5: 553-564. PubMed: 8845746. 8845746 10.1002/pro.5560050401 PMC2143395

[29] Rosenow A, Arrey TN, Bouwman FG, Noben JP, Wabitsch M et al. (2010) Identification of novel human adipocyte secreted proteins by using SGBS cells. J Proteome Res 9: 5389-5401. doi:10.1021/pr100621g. PubMed: 20681635. 20681635 10.1021/pr100621g

[30] Moussa M, Gouranton E, Gleize B+, Yazidi CE, Niot I et al (2011) CD36 is involved in lycopene and lutein uptake by adipocytes and adipose tissue cultures. Mol Nutr Food Res 55: 578-584. doi:10.1002/mnfr.201000399. PubMed: 21462325. 21462325 10.1002/mnfr.201000399

[31] Weikel KA, Chiu CJ, Taylor A (2012) Nutritional modulation of age-related macular degeneration. Mol Aspects Med 33: 318-375. doi:10.1016/j.mam.2012.03.005. PubMed: 22503690. 22503690 10.1016/j.mam.2012.03.005 PMC3392439

